# Developing and translating knowledge for healthy ageing

**DOI:** 10.1016/j.lanwpc.2023.100751

**Published:** 2023-03-27

**Authors:** Hiromasa Okayasu, Wenqian Xu

**Affiliations:** Division of Healthy Environments and Populations at World Health Organization Regional Office for the Western Pacific, Manila, Philippines

The Western Pacific Region continues to experience a sustained change in the age structure of the population. It has one of the largest and fastest-growing older populations in the world. In 2020, there were more than 240 million people over 65 years old living in this region, and this number is predicted to almost double by 2040.^1^

Good health and well-being in older age are vital to individuals and societies. Health is a prerequisite for any social and economic activities, which has become clearer during the COVID-19 pandemic. People with extra years of life in good health can continue to be an integral part of families and communities, thereby strengthening societies.^2^ Good health is also key to the full realization and enjoyment of older people's human rights.

Health is fundamental to all human activities and a driver of sustainable development. Thus, we need a whole-of-society approach to improve population health and transform health systems so that they become an integral part of society (rather than a conventional healthcare delivery system). Evidence shows that investments in health can deliver high social and economic returns.^3^^,^^4^ The initiatives in non-health sectors seeking to address social determinants of health can contribute to healthy ageing and achieving health equity.^5^ Countries should frame their policies and programmes, across the whole of society, to support the optimization of older persons’ functional ability. The health sector can effectively engage other sectors in this effort by building the investment case, drawing on evidence of co-benefits, and deliver benefits for the whole of society by enhancing people-centred integrated care.

To promote healthy ageing, countries should develop a vision based on their social, cultural, economic and political contexts. Countries should develop and drive long-term transformation agendas that reflect their country specific context using foresight-based approaches. For instance, Mongolia has used Health Futures Strategic Dialogues^6^ to engage relevant stakeholders and prepare their health system to meet the future challenges of population ageing through the development of transformation agendas.

Then, countries should plan actions to achieve their desired goals by learning from other countries’ experiences and customizing their approaches to achieve healthy ageing in their particular context. Countries with younger populations can learn from the experience of countries whose populations are already aged in transforming their health and social systems. Aged and super-aged countries can also be inspired by innovative practices and approaches at work in younger/ageing countries. This may include smart care models for older people in China and intergenerational self-help clubs in Viet Nam, for instance. Countries should formulate polices/strategies/plans informed by the best available information and customize programmes/projects based on their contexts.

Countries are strongly encouraged to employ a try-and-learn approach to implementation including at local levels, updating their healthy ageing strategies based on feedback and sharing the knowledge gained with others. Using this flexible approach, policy- and decision-makers can pilot programmes and test the “fit” in a specific local context. With lessons learned, they can expand interventions with proven efficacy to reach more people over a wider geographic area through multi-stakeholder and multi-sectoral collaboration. Given that many countries experience similar challenges related to societal ageing, knowledge sharing can help amplify innovations and solutions to those challenges. Research is a fundamental part of knowledge generation and translation, thus accelerated actions are needed to improve research and data gathering and analysis on healthy ageing.

The WHO Western Pacific Regional Office is committed to supporting Member States to prepare for population ageing. Using a regional “grounds-up” approach, we support countries to identify possible transformative changes needed to achieve the longer-term vision of healthy ageing, customize policies and programmes in their contexts, and update our guidance with country feedback. WHO continues to facilitate research to identify and document best practices in the field of healthy ageing, and promote knowledge sharing in collaboration with governments, scholars, civil society and other stakeholders.

## Declaration of interests

No conflict of interest.
